# Multiscale Modeling of Amyloid Fibrils Formed by Aggregating Peptides Derived from the Amyloidogenic Fragment of the A-Chain of Insulin

**DOI:** 10.3390/ijms222212325

**Published:** 2021-11-15

**Authors:** Michał Koliński, Robert Dec, Wojciech Dzwolak

**Affiliations:** 1 Bioinformatics Laboratory, Mossakowski Medical Research Institute, Polish Academy of Sciences, 5 Pawinskiego St., 02-106 Warsaw, Poland; 2 Faculty of Chemistry, Biological and Chemical Research Center, University of Warsaw, 1 Pasteura St., 02-093 Warsaw, Poland; robert.dec@student.uw.edu.pl (R.D.); wdzwolak@chem.uw.edu.pl (W.D.)

**Keywords:** multiscale modeling, amyloid fibril, flexible docking, fibril structure prediction, peptide aggregation, molecular dynamics, peptide docking, protofilament structure, CABS-dock

## Abstract

Computational prediction of molecular structures of amyloid fibrils remains an exceedingly challenging task. In this work, we propose a multi-scale modeling procedure for the structure prediction of amyloid fibrils formed by the association of ACC_1-13_ aggregation-prone peptides derived from the N-terminal region of insulin’s A-chain. First, a large number of protofilament models composed of five copies of interacting ACC_1-13_ peptides were predicted by application of CABS-dock coarse-grained (CG) docking simulations. Next, the models were reconstructed to all-atom (AA) representations and refined during molecular dynamics (MD) simulations in explicit solvent. The top-scored protofilament models, selected using symmetry criteria, were used for the assembly of long fibril structures. Finally, the amyloid fibril models resulting from the AA MD simulations were compared with atomic force microscopy (AFM) imaging experimental data. The obtained results indicate that the proposed multi-scale modeling procedure is capable of predicting protofilaments with high accuracy and may be applied for structure prediction and analysis of other amyloid fibrils.

## 1. Introduction

The formation of amyloid fibrils, highly ordered β-sheet-rich aggregates of misfolded proteins or peptides, is intimately linked to various degenerative diseases including Parkinson’s disease and diabetes type II [1]. The remarkable thermodynamic and mechanical stability of these entities [2,3], as well as their resistance to digestion by proteolytic enzymes, has made them useful for various organisms; several examples of biologically functional amyloid fibrils have been described in recent years (e.g., [4,5]). Despite several accomplishments in the field, the phenomenon of amyloidogenic self-assembly is not fully understood and certain aspects of amyloid research face significant challenges. The often polymorphic and non-crystallizable character of amyloid fibrils hampers the widespread use of high-resolution structure-determining tools such as solid-state nuclear magnetic resonance (ssNMR) and X-ray diffraction (even though competent applications of ssNMR and X-ray microcrystallography have resulted in a successful determination of structures of various types of amyloid fibrils [6,7,8]). The sheer complexity of conformational transitions leading from singly dispersed protein monomers to an amyloid architecture, with large numbers of molecules involved, and the presence of kinetic traps, pose even bigger challenges to computational studies on amyloid-formation [9,10,11]. Coarse-grained (CG) methods produce simplistic self-assembly models lacking realistic rendering of fine interactions between fibrils and the environment while computational costs of all-atom (AA) molecular dynamics (MD) of realistic systems (i.e., involving many aggregating protein monomers at relatively low concentration in the aqueous environment) are often prohibitive. Thus, MD studies of large amyloid assemblies are rather limited, as the required time scales of such simulations are usually beyond the capacity of the computers accessible to most scientists. Therefore, only small amyloid systems and their local structural rearrangements can be efficiently modeled using AA MD. As physiologically relevant processes of amyloid formation often concern larger and relatively diluted proteins, there remains a significant need for the development of new effective in silico approaches to this problem. An attractive alternative to straightforward AA MD simulations may be a multiscale approach combining fast CG simulations, with MD refinement of the resulting structures. In this way, modeling the evolution of large molecular systems becomes feasible even at prolonged timescales. A plethora of CG approaches has been described earlier, although very few of them provide the necessary resolution and computational acceleration while maintaining an easy connection with more accurate methods [12].

In the multiscale modeling scheme developed for the present study, we used the CABS-dock algorithm [13] supported by appropriate clustering and scoring schemes, followed by AA refinement of the obtained models using MD. The CABS-dock is a tool designed for free docking of peptides to protein receptors. The docking algorithm can treat the protein receptor as a fully flexible object, although its structural fluctuations are usually restricted to an a priori defined surrounding of its known structure. At the same time, some fragments of the protein receptor can be treated as fully flexible, permitting large-scale structural rearrangements induced on the protein/peptide-ligand (or ligands) docking. In the basic version of the CABS-dock algorithm, peptide ligands are treated as fully flexible objects, and the location of the binding site does not need to be specified. In this way, the free docking of a fully flexible peptide ligand to the partially flexible receptor can be simulated. Weak restraints derived from available experimental data can also be easily imposed, making the docking process faster and more precise. The algorithm is very robust, and docking conditions can be easily modified [14].

As a model for our CABS-dock docking simulations, peptide ACC_1-13_—an extremely amyloidogenic N-terminal fragment of the insulin A-chain—was selected [15,16,17]. The ACC_1-13_ peptide comprises the first 13 residues of the A-chain and retains the intrachain Cys6–Cys11 disulfide bond, while the Cys7 residue, involved in the interchain disulfide bond in the parent insulin monomer, is replaced with Ala. We have shown earlier that ACC_1-13_ is the key amyloidogenic region of the larger highly aggregation-prone H-fragment, which is released upon partial digestion of insulin with pepsin [18]. The intact disulfide bond is not only compatible with the formation of a β-sheet structure but also accelerates the process, possibly through the entropic destabilization of free disordered monomers [15]. The disordered state of ACC_1-13_ before the formation of fibrils appears to contribute to the overall aggregation rate by minimizing energy barriers and kinetic traps. In our CABS-dock-based approach, a single ACC_1-13_ monomer was treated as a fully flexible “receptor protein”, whereas the other ACC_1-13_ monomers were treated as “ligands” and were simultaneously docked to the “receptor” peptide. The ligand monomers were also treated as fully flexible molecules, although soft restraints were imposed between pairs of neighboring peptide chains, making their parallel packing preferable. This constrain is due to the following reasoning: the intrachain disulfide bond bends the backbone of ACC_1-13_, and the resulting low symmetry conformation allows the saturation of intermolecular contacts (van der Waals and hydrogen bonds) within the aggregate when the subsequent building blocks are arranged in quasi-translational mode (i.e., in-register parallel strands).

Results of numerous CG docking simulations of multiple copies of ACC_1-13_ peptides were properly clustered and converted into AA representations. Optimization of model geometry and scoring was conducted using AA MD. Next, the obtained protofilament models (by protofilaments we mean peptide assemblies similar in structure to the segments of mature fibrils) were used for the assembly of the final amyloid fibril models, which were then subjected to MD refinement within the explicit solvent environment. Finally, we analyzed the resulting structures of the amyloid fibrils and compared them to the atomic force microscopy (AFM) imaging experimental data. We also briefly discuss the more general meaning of our findings in the context of the structural biology of amyloid fibrils.

## 2. Materials and Methods

### 2.1. Modeling Peptide Aggregation Using CABS-Dock

The CABS-dock standalone version [13] was applied for modeling the peptide aggregation process. This method uses the CABS (C-alpha, beta, and side chains) model [19]. The coarse-graining and discretized representation of modeled structures and its simple and efficient energy calculations enable very fast computations of local moves during Monte Carlo dynamics (that is the basic sampling scheme for the model). The force field of the CABS model consists of several terms, accounting for local structural regularities and specific patterns of interaction characteristics for peptides, proteins, and protein complexes. Such a knowledge-based force field has been derived by statistical analysis of structures accessible in properly sorted PDB [19] entries to avoid overrepresentation of the most frequent folds. The CABS method has proven to be an efficient tool for *de novo* structure prediction [20,21,22], comparative modeling, simulation of protein dynamics, and protein–peptide docking [23,24], including free docking of small proteins to protein receptors [25].

Based on the symmetry argument outlined above in the Introduction, it is assumed that aggregating ACC_1-13_ monomers form amyloid fibrils rich in β-sheet structures, composed of identical parallel-oriented peptide chains. The typical application of the CABS-dock method involves docking of peptide ligands to protein receptors [14]. In the present work, we performed simultaneous and multiple docking simulations of four (or more) identical peptide monomers. All monomers were treated as fully flexible objects and were allowed to bind each other during the docking simulation. To meet the requirements of the CABS-dock modeling scheme, and its input data structure, the first monomer was defined as the receptor molecule, while the remaining monomers were treated as docking ligands. The CABS-dock method requires an initial three-dimensional receptor structure. Therefore, the atomic structure of the peptide chain at its fully extended conformation (ϕ_i_ = ψ_i_ = 180°) was generated using the Avogadro program [26]. This structure, and its amino acid sequence, were used as the receptor input data for the CABS-dock simulation. No intermolecular restraints were defined for “receptor” peptide molecule (the --no-protein-restraints option) allowing for its full flexibility. For the remaining peptides (treated as docked ligands), only their amino acid sequences and secondary structure preferences were provided as the input data. Additionally, during the docking simulation, weak distance restraints (--sc-rest-add option with the distance set to 5 Å and the weight set to 1) were imposed onto the SC (pseudo-atoms representing amino acids side chains in the CABS model [19]) atom pairs of corresponding amino acid residues in adjacent peptide chains forming the fibril. More specifically, restraints were set between pseudo atom SC_j_^i^ and atom SC_j_^i+1^ where *i* is the number of the peptide chain and *j* is the residue number in a particular peptide molecule (a scheme presenting an application of distance restraints used in docking simulations is shown in Figure S1). During the single CABS-dock simulation, 10,000 models were generated in the C-alpha trace representations. For further analysis, 1000 models with the lowest values of interaction energy between docked molecules were selected. Finally, the 10 top-scored models were selected using structural clustering methods. A large number of docking simulations, with different numbers of interacting peptides—including 4, 5 or 6 copies of peptide molecules—were performed.

### 2.2. Test Prediction of Known Protofilament Structures

To validate the aforementioned procedure for modeling peptide aggregation, we performed CABS-dock simulations to test the prediction of three different, already-solved amyloid protofilament structures. For this purpose, the following experimental structures were downloaded from the Protein Data Bank (PDB) database, PDB IDs: 5VOS (11 residue fragments of amyloid-beta A4 protein based on micro electron diffraction) [27]; 2E8D (22 residue fragment of amyloid protofilaments of β_2_-microglobulin based on a combination of ssNMR, X-ray fiber diffraction, and AFM) [28]; and 2MPZ (26 residues structural model of Aβ D23N “Iowa” mutant based on ssNMR, transmission electron microscopy (TEM), and Rosetta model building [29]. During the blind test prediction of selected protofilament structures, the input data contained only the amino acid sequences of the peptide monomer, while all the structural data were treated as unknown. For each of three test systems, 10 independent CABS-dock docking simulations were performed, resulting in 100 top-scored protofilament models. The predicted fibril segments in C-alpha trace representations were then superimposed on experimentally derived structures and compared (in the case of NMR structures, the average C-alpha trace structure, calculated using all available conformations, was used as the reference structure for predicted protofilament models).

### 2.3. Multiscale Modeling Procedure for the Structure Prediction of Amyloid Fibrils

The designed multiscale protocol was used for a prediction of the atomic structure of amyloid fibrils formed by multiple copies of the ACC_1-13_ peptide. The modeling procedure for obtaining smaller fibril segments was analogous to that used in the test prediction of the three experimental amyloid structures outlined above.

**Figure 1:**
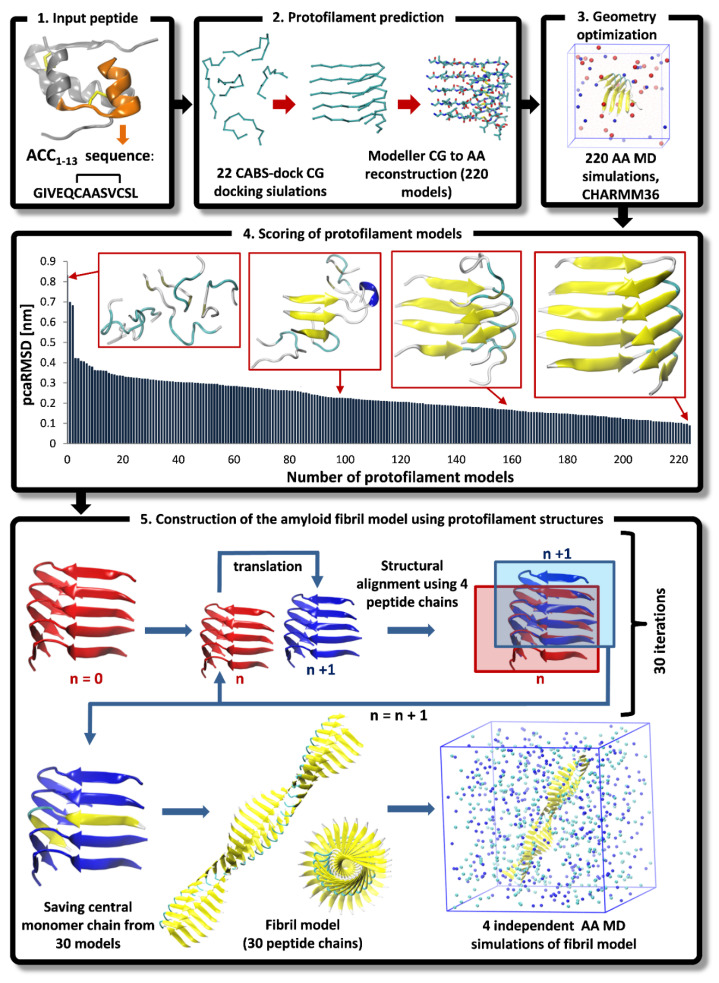
The multiscale modeling procedure for prediction of amyloid fibril structures: (1) preparation of input data for the peptide molecule; (2) prediction of the protofilament structures using CG CABS-dock simulations followed by AA reconstruction; (3) geometry optimization; (4) scoring and selecting the best protofilament models; (5) construction of an amyloid fibril model using the protofilament structures as the building blocks, followed by geometry optimization using AA MD simulations in explicit solvent. For a detailed description of the above procedure see Section 2.3.

In the first step, large numbers of CG models of protofilaments formed by 5 interacting peptide monomers were predicted using the CG CABS-dock method. Then, after reconstruction of AA representations of CG models and MD-based geometry optimization, the refined protofilament structures were used for the assembly of an amyloid fibril model consisting of 30 copies of ACC_1-13_ molecules. The entire multiscale modeling protocol includes 5 major steps which are outlined below and are shown in Figure 1.

1 **Input peptide.** The amino acid sequence of ACC_1-13_ consisting of 13 residues was extracted from the N-terminal part of bovine insulin’s A-chain (PDB ID: 2A3G) [30]. The Cys residue at the 7th position (which is engaged in intramolecular disulfide bonds in the native insulin) was substituted with an Ala residue.
2 **Protofilament prediction.** The CABS-dock program [13] was used for predicting a large number of peptide assemblies formed by 5 interacting monomers (details of docking simulation are described in Section 2.1. During CG docking simulations, additional distance restraints were imposed on pairs of C-alpha atoms of appropriate cysteine residues forming the disulfide bridge in the A-chain of insulin (Cys6-Cys11). The length of these restraints in all peptide monomers was set to 6.5 Å (the average distance observed during the AA MD simulation of a single peptide monomer in explicit solvent). We conducted a total of 22 CG docking simulations, resulting in 220 top-scored protofilament models, each composed of 5 interacting monomers in C-alpha representations. The resulting CG models were converted into their AA representations using the Modeller version 9.25 program [31].
3 **Geometry optimization.** Each protofilament model was inserted into simulation boxes and solvated with 6000 water molecules, 23 Na^+^ ions, and 28 Cl^−^ ions. The system was modeled at a low pH of 1.9; therefore, the side chains of Glu residues were protonated in all peptide chains. Furthermore, the N-termini of all peptide monomers started with a positively charged protonated amino group (-NH_3_^+^), while the C-termini were capped by a neutral protonated carboxyl group (COOH). System equilibration included 20,000 steps of the steepest descent algorithm, followed by 10 ns MD simulations using weak position restraints imposed on the backbone atoms of peptide monomers. This retained the initial conformation of the peptide monomers, allowing for the correct orientation of the surrounding water molecules. Eventually, the production run included unrestrained MD simulations lasting 100 ns. The PME method [32] was used for the treatment of long-range electrostatic interactions. Hydrogen bonds were restrained using the LINC algorithm [33]. The simulation step was 2 fs. The Charmm36 force field [34] parameter set was applied and the TIP3P water model [35] was used for solvent molecules. All simulations were conducted by applying periodic boundary conditions (PBC) at 1013 hPa. The temperature was set to 310 K using the velocity rescale (V-rescale) thermostat. A total of 220 protofilament models extracted from the final frames of the MD trajectories were saved for further analysis.
4 **Scoring of protofilament models.** A straightforward scoring method has been proposed to evaluate and select the most reliable structure of protofilaments for the further construction of amyloid fibril models. Since amyloid fibrils consist of a large number of identical peptide chains forming long stacks of β-sheets with a quasi-translational symmetry along the long fibril axis [23], all peptide monomers in the predicted protofilament model should have very similar chain conformations. We used the symmetry criteria to select the best models of protofilaments. Therefore, for each model, we defined the peptide-chain-average-RMSD parameter (pcaRMSD). The value of pcaRMSD was calculated for each protofilament model using the set of RMSD values obtained during the mutual comparison of all 5 monomers. The RMSD value for each peptide pair was calculated after the structural fitting of two monomers. In this way, the conformations of 5 peptides were compared with each other in a given protofilament model. Low pcaRMSD values correlate with a high level of translational symmetry of monomers within the protofilament structure. The pcaRMSD values were calculated for all 220 models using the following formula:
  (1) $$\mathrm{pcaRMSD}=\frac{1}{M^{2}-M}\overset{M}{\underset{i=1}{\sum }}\overset{M}{\underset{j=1}{\sum }}\sqrt{\frac{1}{N}\overset{N}{\underset{k=1}{\sum }}{\left|X_{k}^{i}-Y_{k}^{j}\right|}^{2}}$$
  where *M* is the number of peptide chains in the protofilament model, *N* is the number of C-alpha atoms, *X_k_* is the coordinate vector for the target C-alpha atom *k*, and *Y_k_* is the coordinate vector for the reference C-alpha atom *k*, whereas *i* and *j* indicate indexes of a particular pair of compared peptide chains in the protofilament model.
5 **Construction of the amyloid fibril model using protofilament structures**. The amyloid fibril model was constructed using the top-scored protofilament structures as building blocks. The fibril assembly procedure included a series of translations. First, two copies of single protofilament models were superimposed using structural alignment based on only four monomer chains in each protofilament model. More specifically, peptide chains from the second to the fifth (monomers 2–5) of the starting protofilament model (Figure 1, protofilament shown in red color) were used for structural fitting of the next identical protofilament model (Figure 1, protofilament shown in blue color), using peptide chains from the first to the fourth (monomers 1–4). During the structural fitting, the coordinates of the starting protofilament model were kept fixed while the second protofilament was translated along the long axis of the formed fibril structure. The thus obtained “translated protofilament” structure was marked with index *n *+* 1* (*n* being the number of translations/procedure iterations), saved after the first cycle of the procedure and used as a starting protofilament structure for the next iteration of the fibril assembly. The procedure was repeated 30 times, resulting in 30 protofilament structures that were consequently translated along the long axis of the predicted fibril. The central peptide chain (chain number 3) was extracted from each of the 30 translated protofilaments and saved. Finally, all extracted central peptides were combined, forming a plausible amyloid fibril model consisting of 30 well-matching peptide chains. The whole modeling procedure was repeated twice (each with 30 iterations) with the two top-scored protofilaments (showing the lowest value of the pcaRMSD parameter) used as a building block, therefore leading to two distinct amyloid fibril models. Both thus obtained fibril models were then subjected to AA MD simulation in explicit solvent. Each system consisted of the fibril model (made of 30 peptide chains), 106,176 water molecules, 420 Cl^-^ ions, and 390 Na^+^ ions. The Simulation box dimensions were 148.1 Å × 148.1 Å × 148.1 Å and in total, the system included 324,618 atoms). At first, each system was equilibrated during the MD simulation with position restraints imposed on the backbone atoms of all peptide chains for the first 100 ns. The production run included unrestrained MD simulations lasting 1 μs and trajectory frames were recorded every 10 ps. For each fibril model, four independent MD simulations were conducted using random initial velocities. Simulation conditions were identical to those used at the earlier step of protofilament optimization (see step 3 **Geometry optimization** of the multi-scale modeling procedure). All AA MD simulations were conducted using the GROMACS version 5.14 program [36]. VMD version 1.9.3 [37] software was used for data analysis and visualization.

### 2.4. Experimental: Evaluation of the β-Sheet Content and Morphology of ACC_1-13_ Amyloid Fibrils

*Samples:* The ACC_1-13_ peptide (sequence: GIVEQCAASVCSL, disulfide bond between residues 6 and 11, no modifications at N- and C-termini) was custom-synthesized by Pepscan (Lelystad, The Netherlands) and provided in the form of trifluoroacetic (TFA) salt, whereas other chemicals used in this study were purchased from Sigma-Aldrich (St. Louis, MO, USA). The purity of the peptide was assessed by the supplier using MS—UPLC analysis (mass spectrometry coupled to ultra-performance liquid chromatography) and exceeded 96%. The stock freeze-dried peptide was solubilized according to the previously described protocol [15], consisting of dispersion of solid pellets in 8M guanidine hydrochloride (GdnHCl) solution, pH 9.0, at a 6 mg/mL peptide concentration followed by 30 min incubation at room temperature. The clear peptide solution was centrifuged at 13,400 rpm for 5 min to remove traces of insoluble matter. This initial centrifugation procedure was taken as a precaution to remove bubbles and traces of insoluble material, even though all the peptide solutions in concentrated GdnHCl were clear and homogenous prior to the centrifugation. Hence, any decrease in peptide concentration due to centrifugation was negligible. The sample was diluted with an appropriate volume of 60 mM NaCl, pH 1.9 also containing the amyloid-specific fluorophore: Thioflavin T (ThT); after mixing, the pH was re-adjusted to 1.9. This solution was swiftly diluted with an acidified (pH 1.9) aqueous solution of GdnHCl, NaCl, and ThT to obtain samples for the kinetic experiment in which the de novo fibrillization of ACC_1-13_ was monitored. All samples contained ACC_1-13_ peptide at the specified concentrations (range between 0.2 and 1.0 mg/mL) dissolved in 1.33M GdnHCl, 50 mM NaCl, 20µM ThT, and H_2_O, at pH 1.9.

*Fibrillization kinetics:* For ThT-fluorescence-based measurements (excitation/emission parameters of ThT: λ ex. 440 nm/λ em. 485 nm) of ACC_1-13_ fibrillization kinetics, a CLARIOstar^®^ plate reader from BMG LABTECH (Offenburg, Germany) and 96-well black microplates were used. Typically, wells were filled with 150 µL volumes of the diluted peptide samples specified above. For each peptide concentration case, three independent kinetic trajectories were collected (the error bars correspond to the standard deviations). Measurements were carried out at 37 °C and 300 rpm agitation. After this, the kinetic experiment samples of aggregated ACC_1-13_ were collected from the plate reader and subjected to conformational and morphological analysis.

*Conformational analysis (estimation of β-sheet content):* Aqueous suspensions of aggregated ACC_1-13_ scatter light strongly, making measurements of circular dichroism spectra in the far-UV region problematic. Instead, we have employed infrared absorption spectroscopy (in the conformation-sensitive amide I band frequency region), which is far less susceptible to artifacts stemming from light-scattering. To this end, we have used a Nicolet iS50 FT-IR (Fourier transform infrared) spectrometer from (Thermo Fisher Scientific (Waltham, MA, USA) equipped with a single-reflection diamond attenuated total reflectance (ATR) accessory and a DTGS detector. Liquid samples of aqueous suspensions of fibrils collected after the kinetic experiment were centrifuged, and subsequently washed extensively with acidified water and trans-ferred onto the diamond surface of the ATR accessory and gently dried in situ. Subsequently, infrared spectra of the thus obtained films were collected. For a single spectrum, 32 interferograms of 2 cm^−1^ resolution were co-added. Standard ATR correction was carried out [38]. The data processing and peak-fitting procedure were conducted using GRAMS software (Thermo, Waltham, MA, USA). The molar absorptivities in the amide I band region of various secondary structure types used for the estimation of β-sheet content were obtained from the work of de Jongh et al. [39].

*Morphological analysis:* Aggregates of ACC_1-13_ formed during the kinetic measurements were subjected to atomic force microscopy (AFM) imaging. A small portion of suspension collected after the completion of the kinetic experiment was centrifuged, washed with acidified water to remove excess salts, and subsequently diluted 5 times. A 10 μL portion of the suspension was swiftly transferred onto freshly cleaved mica and left to dry overnight. AFM tapping-mode measurements were carried out using a Nanoscope III atomic force microscope from Veeco Instruments (Plainview, NY, USA) and TAP300-Al sensors (res. frequency 300 kHz) from BudgetSensors (Sofia, Bulgaria). The cross-sections were obtained from the corresponding height images.

## 3. Results and Discussion

### 3.1. Test Prediction of Fibrils with Known Structures

**Figure 2:**
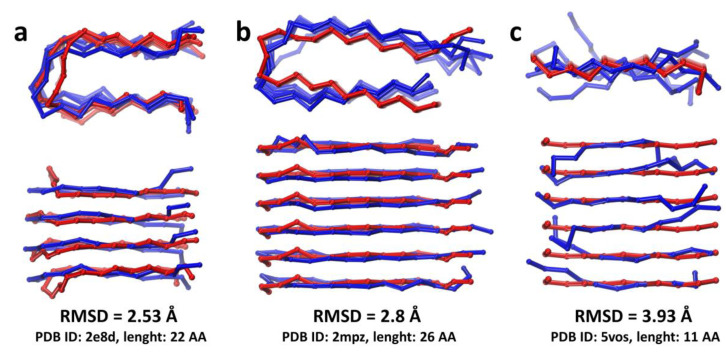
Predicted models of the three fibril structures. The model with the lowest RMSD value obtained from the set of 100 top-scored models is shown in C-alpha trace representation (color blue) and superimposed onto the reference experimental structure (color red) for each fibril: (**a**) 2e8d, (**b**) 2mpz and (**c**) 5vos. Fibril structures are shown from two different perspectives: along the long axis of the fibril (upper panel), and the side view (lower panel). At the bottom of each panel, the following information is included: RMSD value calculated for the predicted fibril, PDB ID of the reference structure, and the number of amino acid residues in the single peptide chain.

To assess the accuracy of the CABS-dock method for the prediction of protofilament structures assembled from short peptides, we selected three fibrils with known experimentally resolved structures deposited in the PDB database. These particular fibril structures were chosen using the following three criteria: (i) the length of the monomeric peptide chain forming the fibril should have similar length to the ACC_1-13_ peptide (~13 residues); (ii) the fibril structure should consist of parallel peptide chains, (iii) the copies of the monomeric peptides molecules should adopt similar conformations upon assembly of fibrils. It should be emphasized that the only input data for the test simulations consisted of amino acid sequences and the number of peptide chains forming an aggregate. Ten independent docking simulations were performed for each system, resulting in a total of 100 top-scored models in C-alpha trace representation for each of three fibrils. Finally, predicted models were compared with experimental structures. The models with the lowest RMSD values (calculated for C-alpha atoms) are shown in Figure 2.

The obtained fibril models reveal a high degree of similarity to the reference experimentally determined structures. During simulations of the aggregation processes, the two longest peptides (see Figure 2, panels a and b) formed U-turns approximately in the middle of the peptide chains. This led to the assembly of two parallel β-sheets in the both predicted structures. Low values of RMSD, below 3 Å, reflect a good accuracy of predicted structures. The model of the third fibril formed by the shortest peptide (see Figure 2, panel c) was less accurate, with an RMSD value of 3.93 Å. The interacting peptides adopted an extended conformation, but due to the local distortions and poor symmetry of adjacent peptide chains, the β-sheet could only be formed in fragments of the created fibril model.

These results of the test predictions demonstrate that the CABS-dock CG docking method is capable of predicting such amyloid structures with good accuracy. However, it needs to be stressed that the presented models were the best (with the lowest values of RMSD when compared to the reference structure) found in the set of 100 top-scored structures resulting from 10 independent docking simulations for each system. The identification of the most accurate structure in the generated model set of top-scored structures is always a challenging task [14,23,24], and many different scoring methods have been developed for such a purpose [12,40].

### 3.2. Amyloid Fibril Models Predicted for Insulin-Derived ACC_1-13_ Peptide

The multiscale modeling procedure designed in this work was used for the prediction of the three-dimensional structure of amyloid fibrils formed by 30 copies of interacting ACC_1-13_ monomers. It is assumed that the amyloid fibril is composed of in-register parallel identical peptide chains. The CABS-dock method was used to simulate the aggregation process, in which five identical copies of peptide formed a large number of protofilaments. Finally, the two top-scored protofilament models, selected by symmetry criteria, were used for the construction of target amyloid fibril models.

AA simulation of the self-assembly of amyloid fibril structures for this or a similar system is beyond the capabilities of contemporary MD modeling tools. Various factors contribute to such limitations, including the slow diffusion of interacting peptide monomers over long distances at physiologically relevant concentrations and large conformational changes occurring during amyloid fibril self-assembly [41]. Here, we used the CABS-dock method for CG simulation of the aggregation process of five copies of ACC_1-13_ peptide (each consisting of 13 amino acid residues).

The CABS-dock method has proven to be a very efficient and versatile tool for modeling a wide range of protein and peptide systems [12,14], including protein–peptide docking [13,14], docking of peptides to membrane receptors [23], peptide docking with large structural changes of the protein receptor and disordered structures [42,43] and also identification of peptide cleavage sites for protease–substrate systems [24]. We also showed above that this method is effective for the prediction of protofilaments formed by even longer peptides (with more than 20 amino acid residues). The most accurate protofilament structures found in the 100 top-scored models (resulting from 10 independent docking simulations) were close to the experimental structures (RMSD values calculated for C-alpha atoms were below 3 Å).

We have modified the tested procedure to further improve its accuracy for modeling the aggregation of insulin-derived peptide chains. First of all, we have increased the number of docking simulations to 22 for the modeled system, resulting in 220 top-scored models. We have also performed an additional optimization of the geometry of all predicted protofilament models by conducting 100 ns of unrestrained AA MD in explicit solvent. In our opinion, this procedure is more than sufficient for obtaining reliable models of protofilaments composed of peptides of this size. It should also be emphasized that the ACC_1-13_ peptide is shorter than the two peptides used for the test predictions and, importantly, that it has an internal disulfide bond that greatly reduces the flexibility of the peptide chain and limits the conformational space that must be sampled. Of course, this internal disulfide bond was taken into account during the docking simulations.

The pcaRMSD parameter values measured for the resulting 220 protofilament models after AA MD optimization ranged from 0.88 Å to 8.3 Å. The analysis of the obtained structures showed that the pcaRMSD parameter (see Equation (1)) strongly correlated with the symmetry of the interacting peptide chains in the protofilament models. The structures of the four models with different pcaRMSD values are shown in Figure 1, in the panel presenting the fourth step of the modeling procedure. The two top-scored protofilament models (presenting the lowest pcaRMSD values) with the highest symmetry were selected for the assembly of the fibril models. Even though these two models were obtained via different initial CABS-dock runs, followed by independent AA MD simulations, they show surprisingly high structural similarity. The RMSD value calculated for the C-alpha atoms after structural fitting of the final models was below 1 Å (Figure S2). This shows the importance of the AA MD geometry optimization step which was applied after reconstructing the CG models to AA representations. First of all, even small conformational changes in the peptide backbone occurring during MD simulation may significantly improve the packing of amino acid side chains, leading to the formation of stable β-sheets. Secondly, the stability of the protofilament structure can also be verified during the MD simulation (e.g., a weakly bound peptide can dissociate from the protofilament structure). The importance of MD-based optimization was observed in the case of the two selected top-scored protofilament models, where the two initial models (AA structures resulting from the reconstruction of the CG models before MD optimization step) showed noticeable structural differences (RMSD = 3.95 A). During the MD simulations, both models converged to nearly identical structures with high symmetry and a high β-sheet content (see Figure S2).

**Figure 3:**
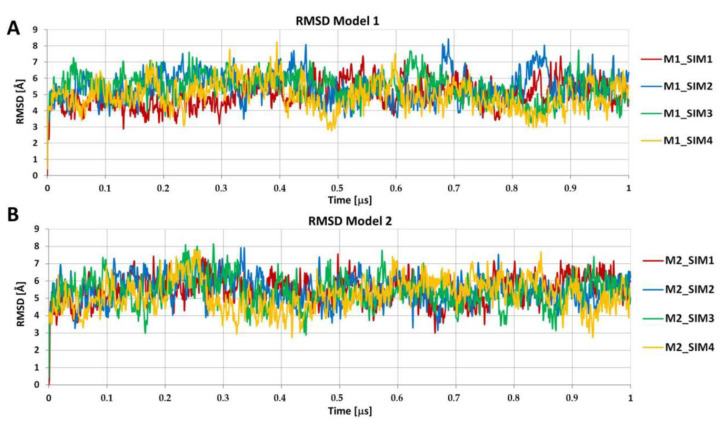
Plots showing the RMSD values for C-alpha atoms of amyloid fibrils calculated during AA MD simulations lasting 1 μs. For each of the two amyloid fibril models (model 1—panel (**A**), and model 2—panel (**B**)), 4 independent MD simulations were conducted using random starting velocities.

The two fibril models were assembled using the two selected protofilament structures during a series of structural alignments and translations along the long axis of the created fibril (for details of the modeling procedure see Figure 1, Step 5, and Section 2.3). The two resulting fibril structures, composed of 30 copies of interacting ACC_1-13_ peptide monomers, were subjected to 1 μs unrestrained AA MD simulations. In total, four independent simulations were conducted for each fibril model, resulting in eight amyloid fibril structures. During the MD simulations, the fibrils adopted stable and intact structures, and no single ACC_1-13_ peptide monomer detached from the rest of the structure. The RMSD values monitored during the simulation, calculated for C-alpha atoms, ranged from 3 Å to 8 Å (Figure 3). The fluctuations of RMSD values (reaching around ~8 Å) were related to the thermal motions of the long fibril and its bending, which is consistent with the dynamic nature and flexibility of such long and thick structures. Fluctuations of the simulated amyloid fibril are shown in Figure S3, based on the data for the MD simulation of the 1st fibril model (M1_SIM1). For this purpose, 1000 fibril structures were extracted from a 1 μs trajectory, with a 1 ns time interval between recorded frames. Subsequently, the extracted structures were superimposed on each other and displayed in C-alpha trace representations.

**Figure 4:**
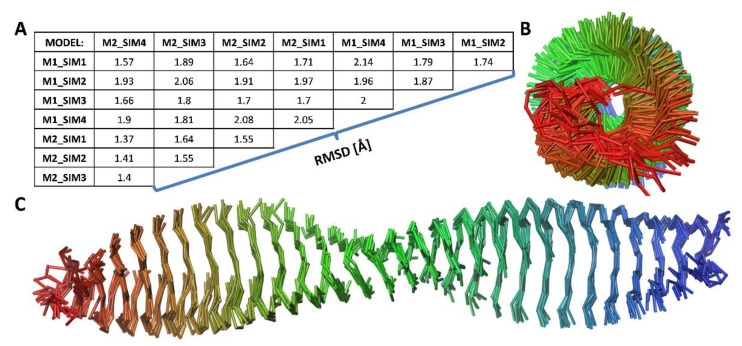
Comparison of predicted amyloid fibrils. The structures of 8 models were extracted from the MD trajectories (frames recorded during the last 500 ns of MD simulation were treated as a single cluster, and the central cluster structure was selected as the final model for each trajectory). The RMSD values for all model pairs were calculated after superimposing every two models and stored in the matrix—panel (**A**). All 8 resulting models superimposed on each other in C-alpha trace representations are shown in panel (**B**) (view along the long axis of the fibril) and panel (**C**) (side view of the fibril). For clarity, the subsequent monomers (from 1 to 30) in the fibril models were marked with different colors from red to blue.

To compare final amyloid fibril models, we have extracted representative structures from eight MD trajectories. For this purpose, all frames recorded during the last 500 ns of each 1 μs MD simulation were treated as a single cluster of fibril structures and the central cluster structure (centroid) was selected as a representative fibril model. Superimposition of the eight selected models showed a remarkable structural similarity of the simulated fibrils (Figure 4) for such relatively thin and long structures. Indeed, the RMSD values calculated during the mutual comparison between all the fibril models were below 2 Å for most of the analyzed cases (see RMSD matrix in Figure 4, panel A). We also used the PROCHECK program to analyze the structure of the amyloid fibrils; Ramachandran plots for all eight extracted models are included in the Supplementary Materials (Figure S4).

We used frames extracted from the trajectories recorded during the MD simulations to analyze the structural properties of the modeled fibrils. We measured the length of the modeled fibril as the distance between the center of mass of the first and last peptide in the simulated fibril model (see Table 1). The length was oscillating around 134 Å and was statistically identical for all models. Next, we measured the mean rotation angle between the two adjacent ACC_1-13_ chains in the fibril model. For this purpose, we defined a line segment in each monomer chain connecting the C-alpha atoms of the Val3 and Ser9 residues. The fragment of the peptide chain between these two residues was also engaged in β-sheet formation and adopted an extended conformation. The line segments were then projected onto a plane perpendicular to the long axis of the amyloid fibril. The angles between the projected line segments for all the adjacent monomers were measured and the mean values were calculated for each conducted simulation (see Table 1). The values of the rotation angle were observed in the range from 8.9 ± 0.67 to 9.16 ± 0.72 degrees. Based on the calculated length of the amyloid fibril composed of 30 ACC_1-13_ molecules and the mean rotation angle, we can estimate the helical pitch value for about 17.9 nm (which accounts for about 40 peptide monomers).

**Figure 5:**
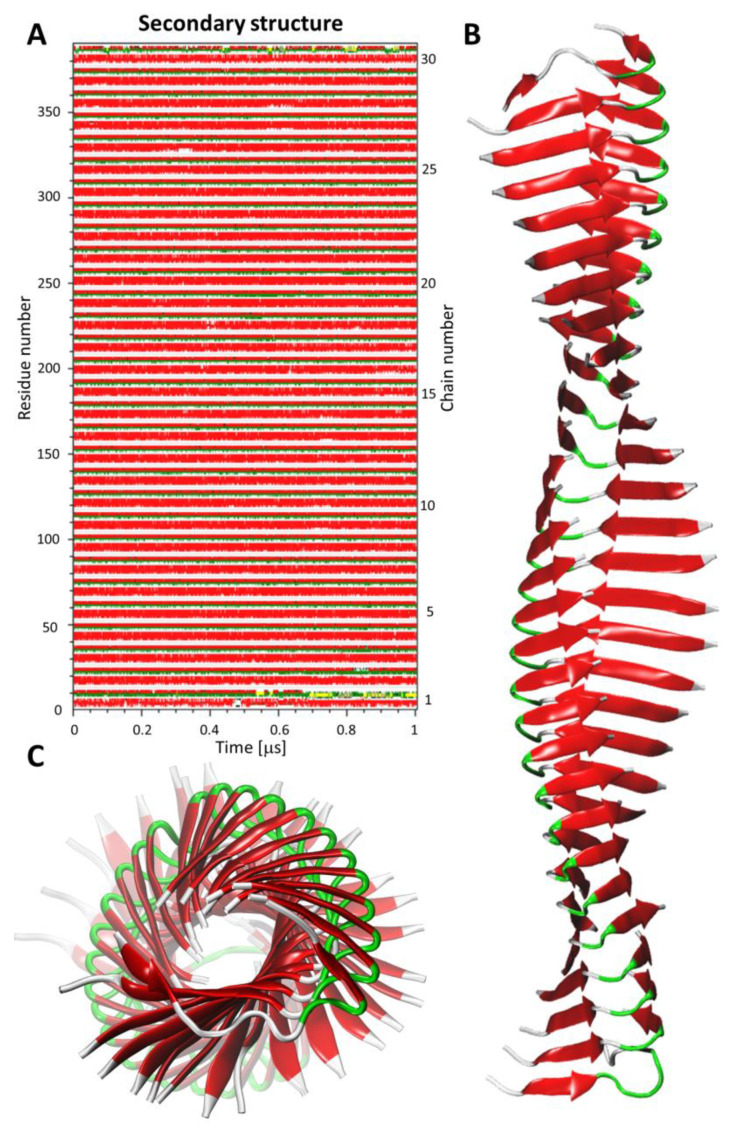
Secondary structure evolution plot calculated for the model of the amyloid fibril (M1_SIM1) during 1 μs AA MD simulation—panel (**A**). Snapshot of the simulated amyloid fibril model in a cartoon representation: side view—panel (**B**), and view along fibril long axis—panel (**C**). Secondary structure assignment was carried out using DSSP software [44]. The following coloring scheme was used: coil—white, β-sheet—red, β-bridge—black, turn—green, bend—yellow, α-helix—blue, 3_10_-helix—grey.

The conservation of the β-sheet content in the predicted models was measured using the DSSP [44] method based on all recorded frames. On average, almost 50% of the ACC_1-13_ residues were engaged in the H-bond network, forming β-sheets (see Table 1). The plot presenting the conservation of the secondary structure during MD simulation and the resulting model of the amyloid fibril in a cartoon representation is shown in Figure 5.

The two β-sheets are separated by a short U-turn formed by the Ala8 and Ser9 residues. The geometry of the peptide monomer chain and β-sheet orientation are stabilized by the disulfide bond formed by Cys6 and Cys11, respectively (see Figure S5). The backbone of the monomer forms a cavity between the two β-sheets. The cavity is occupied by tightly packed side chains of Ala8 and Leu13 residues, preventing the penetration of water molecules. The stick model presenting the structure of a single monomer in the predicted fibril model is shown in Figure S5.

### 3.3. β-Sheet Content in Predicted Fibril Models—Comparison to Experimental Data

**Figure 6:**
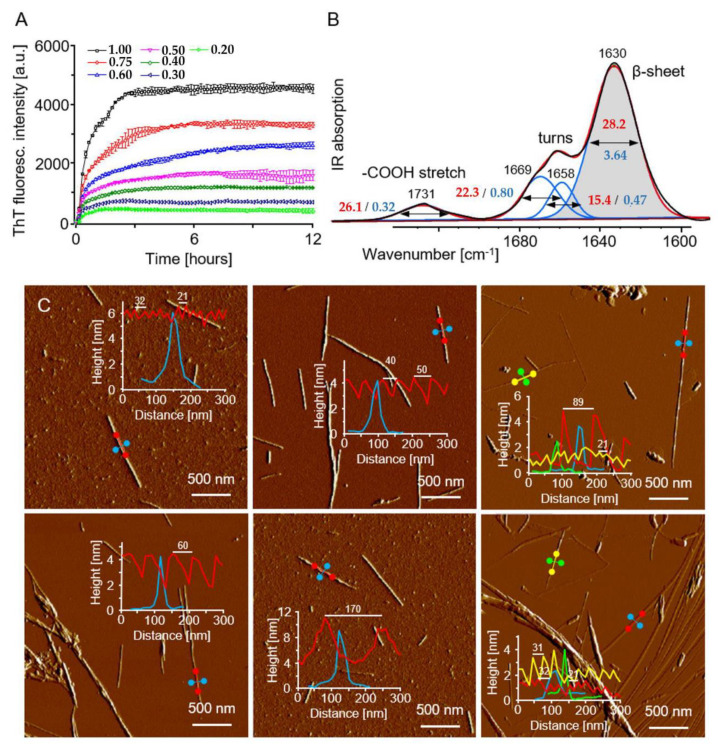
Experimental characteristics of the amyloidogenic ACC_1-13_ peptide. (**A**) Kinetics of de novo fibrillization of ACC_1-13_ at various concentrations ([mg/mL]) probed with a ThT fluorescence assay at pH 1.9 and 37 °C. (**B**) Infrared absorption of ACC_1-13_ fibrils in the amide I band region: the original spectrum (black contour) and peak-fitting with Gaussian components (individual in blue, summation in red), values of FWHM and the integral intensity of component bands are indicated in blue and red, respectively. (**C**) AFM amplitude images of various amyloid specimens of ACC_1-13_. Superimposed cross-sections reveal the height (diameter) and periodicity of fibrils. In each AFM image, the same colors were used to indicate analyzed fibrillar specimen and the obtained cross-section curve.

Certain aspects of the predicted structures of ACC_1-13_ fibrils, such as β-sheet content, diameter, and periodicity of the twisted surface (helical pitch), can, in principle, be compared with easily accessible experimental data. Hence, we have carried out *de novo* fibrillization of ACC_1-13_ according to the established protocol [15]. The kinetic trajectories reflecting the rapid growth of amyloid mass shown in Figure 6A were obtained using fluorescence of Thioflavin T (ThT), an amyloid-specific molecular rotor whose quantum yield of fluorescence increases by 2–3 orders of magnitude upon intercalation onto the amyloid surface [45]. We note that even under these strictly *de novo* conditions, aggregation is very fast, with a practically undetectable lag phase. Aggregates collected at the end of the kinetic experiment were subjected to conformational and morphological analysis. Strong light scattering on precipitating ACC_1-13_ fibrils hampered the acquisition of high-quality circular dichroism spectra. However, we were able to use infrared spectroscopy to estimate β-sheet content through the deconvolution of the conformation-sensitive amide I vibrational band by taking advantage of the fact that different components of the secondary structure absorb infrared light of distinct frequencies. The infrared spectrum of the ACC_1-13_ amyloid fibrils shown in Figure 6B encompasses both the amide I band (frequency range 1600–1690 cm^−1^) and the minor band at 1731 cm^−1^ corresponding to protonated carboxyl groups. The amide I band is clearly dominated by a single spectral component at 1630 cm^−1^ assigned to the canonical parallel β-sheet structure often found in amyloid fibrils [46], and is flanked by smaller bands at and above 1660 cm^−1^ that are assigned to turns. While this band could, in principle, be overlapped to a degree by IR absorption from traces of TFA present in the commercial peptide, the similarity of the spectral contour to that of the insulin fibrils formed in the total absence of TFA [18] and the absence of other infrared signature features of TFA suggest that this problem is negligible. Based on peak-fitting with Gaussian functions, the integral intensity of the 1630 cm^−1^ β-sheet component amounts to 74% of the total amide I band intensity. However, this should not be interpreted directly as the percent of this conformation. Taking into account the fact that the vibrational molar extinction coefficient of the β-sheet is approximately twice as much as that of turns [39] the corrected estimate of this structure is approximately 59%.

We have subsequently used AFM to probe the morphologies of fibrillar ACC_1-13_. In Figure 6C, amplitude images of various amyloid fibrils found in the *de novo* aggregated samples are presented. Overlaid are cross-sections obtained from the corresponding height images. The cross-sections were obtained in both directions: along the long fibrillar axis (reflecting the periodicity of the fibril’s twist—the helical pitch), and in the perpendicular direction (essentially showing the fibril’s diameter). The images indicate very clearly that amyloidal ACC_1-13_ is polymorphic on a morphological level. The values for the diameters of the amyloid specimen vary in the range between 2 and 8 nm, suggesting that most of these fibers consist of multiple individual hierarchically intertwined protofilaments, which is a common self-assembly pattern found in amyloid aggregates [47,48]. The corresponding periodicities also vary significantly between 21 and 170 nm. It appears that for most of these specimens, the increasing periodicity correlates with the increasing diameter, which is rather intuitive [49]. While the AFM allowed us to capture the helical twists of the ACC_1-13_ fibrils, given the observed high diameters, those most likely correspond to higher order structures rather than individual protofilaments simulated in this study. On the other hand, some of the thinnest specimens (~2 nm in diameter) had a rather low helical pitch (31–32 nm), which still exceeded the pitch values obtained for single protofilaments in the in silico part of this study. It is unclear to us whether this discrepancy arises from the fact that, again, these thin specimens are not individual but doubly intertwined protofilaments, or from the inherent limitations of the AFM method. Specifically, one has to take into account the fact that amyloid examined with an AFM probe is deposited on a negatively charged mica surface [50] whereas ACC_1-13_ is positively charged at low pHs, at which fibrils are formed. Hence the coulombic attraction to the surface could result in a structural deformation (‘flattening’) of fibrils, as the higher periodicity increases the effective protein–mica close contact area. We argue that this effect may have a strong impact that increases the helical pitch value of the ACC_1-13_ amyloid deposited on mica vis a vis the computationally studied isotropic conditions.

## 4. Conclusions

We have designed and tested a new multiscale protocol for the simulation of amyloid fibril assembly. The protocol starts from multiple CG simulations of mutual docking of peptides forming protofilament structures. The most plausible structures of these assemblies are selected by the ranking and clustering algorithms. The obtained protofilament models are reconstructed to AA representations and their structures are optimized during AA MD simulations. Models showing the highest symmetries are used for building the larger fibril structures in the AA representations. Finally, the amyloid fibril structures are refined and analyzed during AA MD simulations in explicit solvent. For several reasons, the resulting models appear to be surprisingly reliable. The credibility of the proposed docking protocol has been validated by the test prediction of known protofilament structures. During prediction, only peptide sequences were used as the input data for the CABS-dock algorithm. This is a strong test of the docking stage.

The final structures of the amyloid fibril composed of ACC_1-13_ peptides are in reasonable agreement with the experimental data, both in terms of estimated β-sheet content and the observed helical pitch length. We believe that the work reported here demonstrates the appealing potential of multiscale modeling of viable amyloid fibrillary structures which are not accessible to the straightforward MD approaches. The multiscale modeling procedure designed in this work is versatile and could also be used for structure prediction and analysis of other biologically important amyloid fibrils created by in-register parallel peptide monomers.

## Figures and Tables

**Table 1:** Structural properties of the obtained models of amyloid fibrils measured during AA MD simulation.

Model	d [Å] ^a^	α [Deg] ^b^	% Res. with H-Bonds ^c^	No. Res. with H-Bonds ^d^
M1_SIM1	134.13 ± 2.33	9.15 ± 0.61	49.92 ± 1.21	194.7 ± 4.72
M1_SIM2	133.32 ± 2.21	9.06 ± 0.72	50.23 ± 1.22	195.9 ± 4.78
M1_SIM3	133.94 ± 2.27	9.02 ± 0.68	49.85 ± 2	194.42 ± 7.81
M1_SIM4	134.14 ± 2.34	9.16 ± 0.72	49.18 ± 1.4	191.82 ± 5.46
M2_SIM1	133.76 ± 2.25	9.03 ± 0.68	49.92 ± 1.17	194.69 ± 4.6
M2_SIM2	134.4 ± 2.37	8.9 ± 0.67	49.73 ± 1.96	193.98 ± 7.67
M2_SIM3	134.3 ± 2.32	9.16 ± 0.69	49.58 ± 1.22	193.37 ± 4.77
M2_SIM4	134.23 ± 2.34	9.16 ± 0.68	49.48 ± 1.24	192.99 ± 4.84

^a^ The distance, measured along the long axis of the amyloid fibril, between the center of mass of the first peptide chain and the center of mass of the last peptide chain. ^b^ The angle measured between the adjacent peptide chains in the amyloid fiber. ^c^ The percentage of amino acid residues involved in the formation of H-bonds. ^d^ The number of amino acid residues involved in the formation of H-bonds.

## Supplementary Materials

The following are available online at https://www.mdpi.com/article/10.3390/ijms222212325/s1.
