# Supplementary material for: Multiscale Modeling of Amyloid Fibrils Formed by Aggregating Peptides Derived from the Amyloidogenic Fragment of the A-Chain of Insulin
Source: Int J Mol Sci. 2021 Nov 15;22(22):12325. doi: 10.3390/ijms222212325 (PMC8621111; doi:10.3390/ijms222212325)
Supplement: Supplementary file 1 [file ijms-22-12325-s001.zip › ijms-1442549-supplementary.pdf]

Supporting information

Multiscale Modeling of Amyloid Fibrils Formed by Aggregating Peptides Derived from the Amyloidogenic Fragment of the A-Chain of Insulin

Michał Koliński[1*], Robert Dec[2], Wojciech Dzwolak[2]

[1] Bioinformatics Laboratory, Mossakowski Medical Research Institute, Polish Academy of Sciences, 5 Pawinskiego St., 02-106 Warsaw, Poland; mkolinski@imdik.pan.pl
[2] Faculty of Chemistry, Biological and Chemical Research Center, University of Warsaw, 1 Pasteura St., 02-093 Warsaw, Poland; robert.dec@student.uw.edu.pl (R.D.); wdzwolak@chem.uw.edu.pl (W.D.)

** Correspondence: mkolinski@imdik.pan.pl*

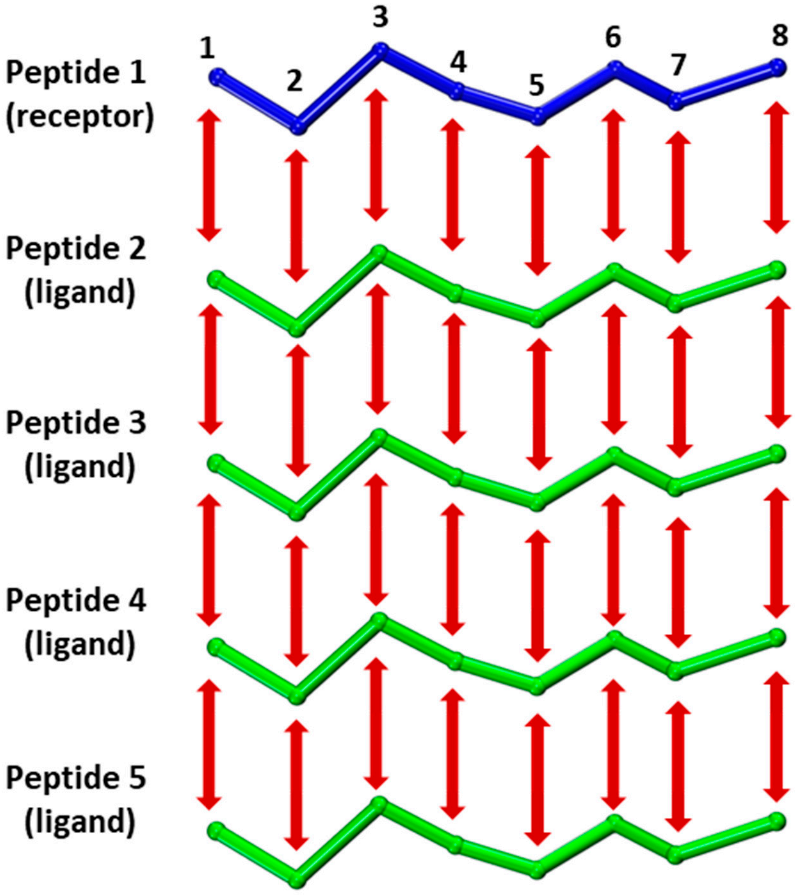

Figure S1. Graphical representation of the distance restraints used during peptide CG docking simulations. The red arrows indicate the distance restraints imposed on the SC atoms pairs of the corresponding amino acid residues in adjacent peptide monomers in the predicted protofilament model. The restraint distance was set at 5 Å. In the example above, five peptide chains (with 8 amino acid residues in the peptide chain) are docked simultaneously during a single simulation. According to the CABS-dock convention, the first peptide chain is treated as the receptor protein (blue color). The remaining peptide chains are treated as docked ligands (green color).

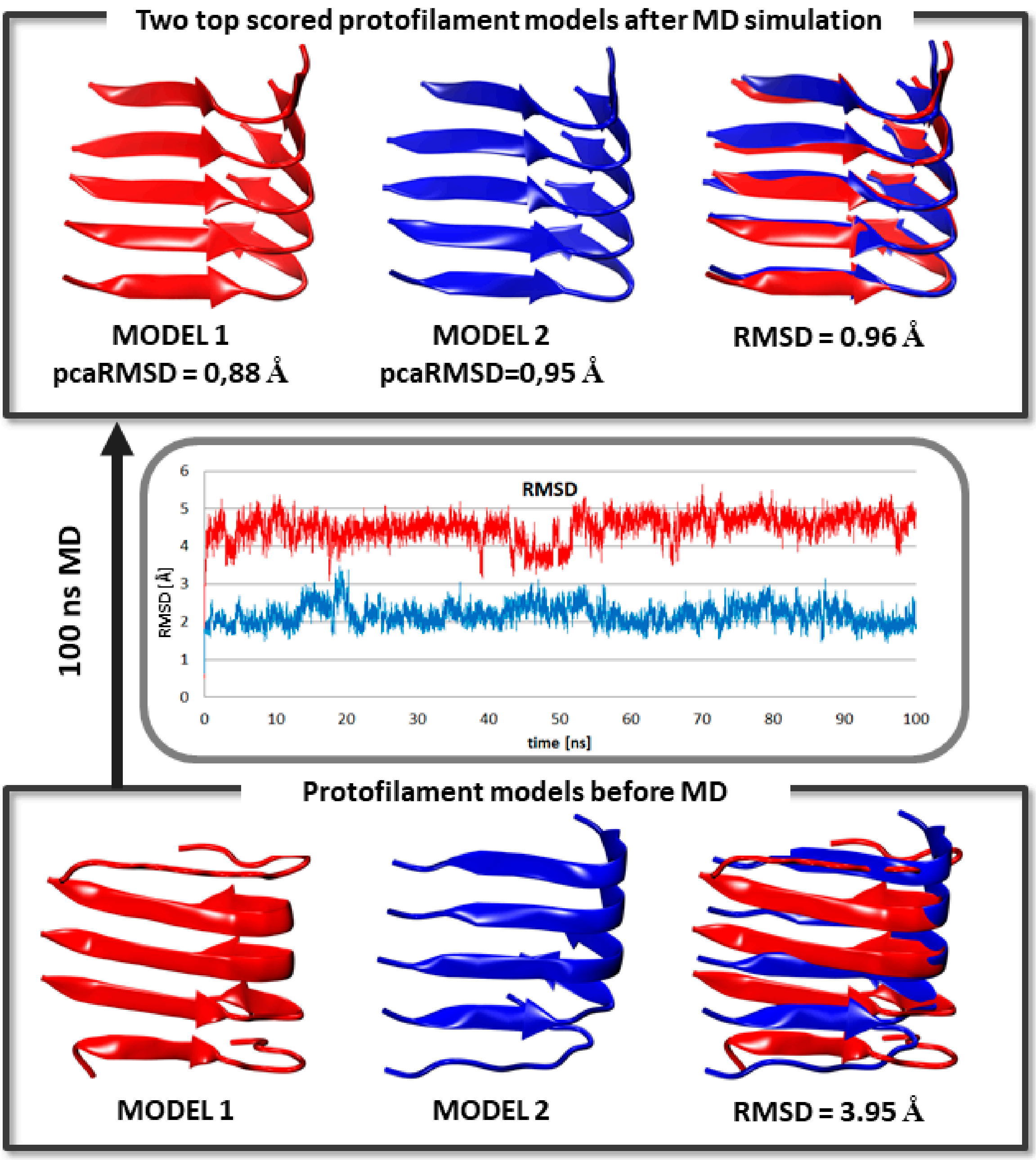

Figure S2. The two top-scored models of protofilament structures consisting of 5 peptide chains. Two models with the lowest pcaRMSD value after MD geometry optimization were selected. The top panel shows two models after MD simulation, bottom panel shows the same two models before MD simulation. On the right side, two models are superimposed on each other. The middle panel shows a plot of the RMSD values recorded during 100 ns AA MD simulation of the two protofilament models.

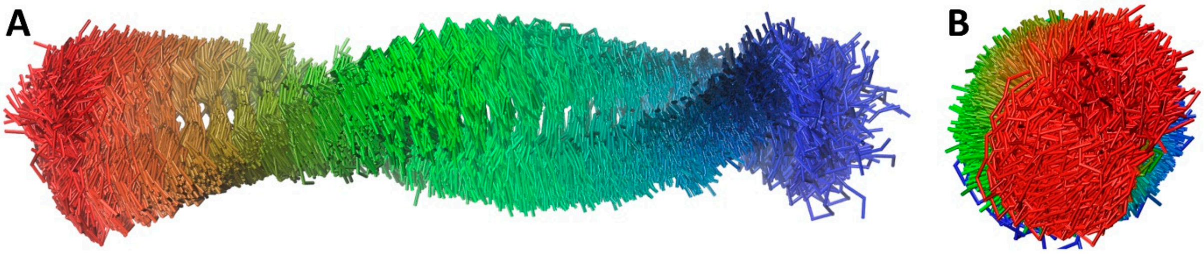

Figure S3. Amyloid fibril structure fluctuations during MD simulation. Fibril structures were extracted every 1 ns of the 1μ trajectory. Next, 1000 extracted structures were superimposed on each other and displayed in C-alpha trace representation. The fibrils are shown in a side view - panel A, and a view along the long axis of the fibril. The data was extracted from the simulation 1 of the fibril model 1 (M1_SIM1).

PROCHECK

Ramachandran Plot

MODEL 1

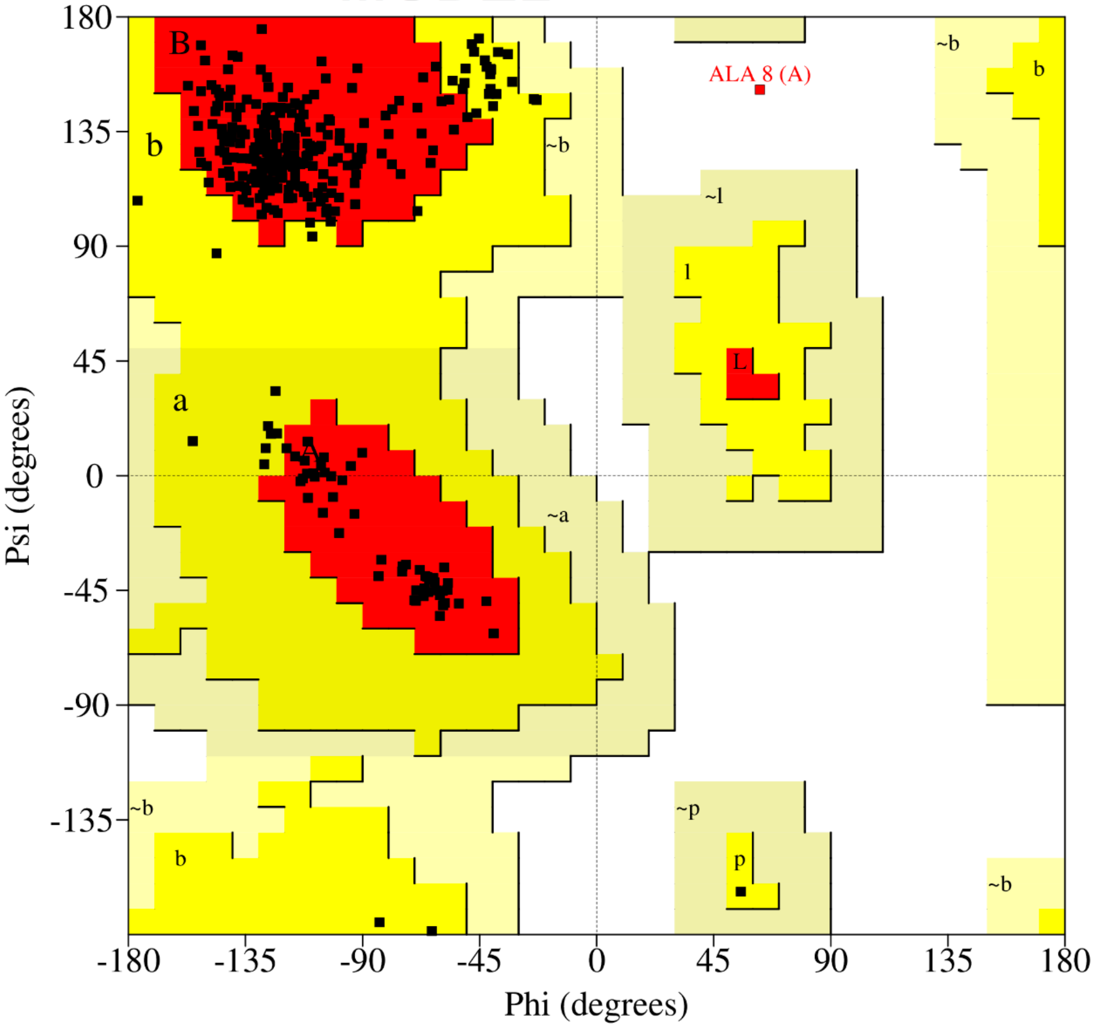

Plot statistics

Residues in most favoured regions [A,B,L]	290	87.9%
Residues in additional allowed regions [a,b,l,p]	39	11.8%
Residues in generously allowed regions [~a,~b,~l,~p]	0	0.0%
Residues in disallowed regions	1	0.3%
	----	------
Number of non-glycine and non-proline residues	330	100.0%
Number of end-residues (excl. Gly and Pro)	30	
Number of glycine residues (shown as triangles)	30	
Number of proline residues	0	

Total number of residues	390	

PROCHECK

Ramachandran Plot

MODEL 2

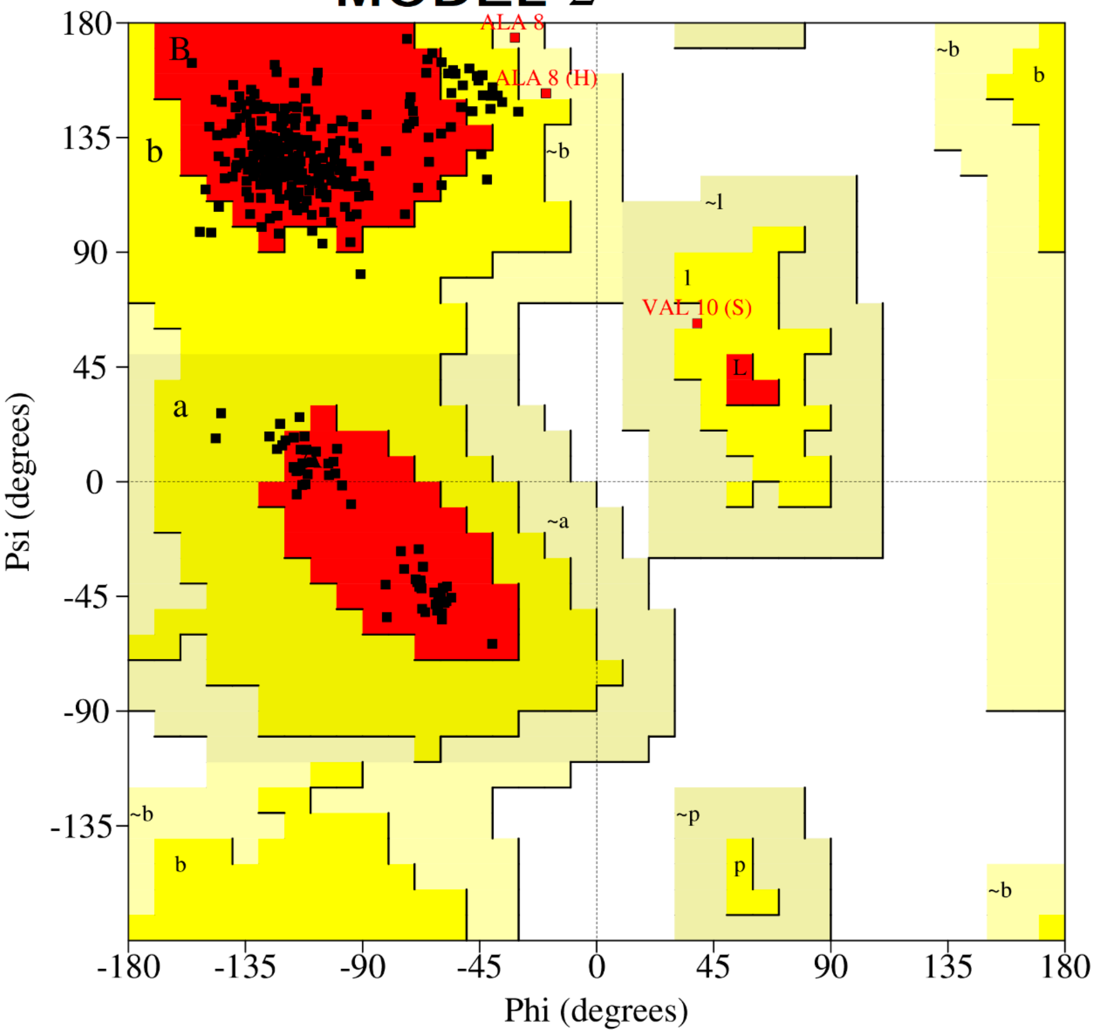

Plot statistics

Residues in most favoured regions [A,B,L]	292	88.5%
Residues in additional allowed regions [a,b,l,p]	35	10.6%
Residues in generously allowed regions [~a,~b,~l,~p]	3	0.9%
Residues in disallowed regions	0	0.0%
	----	------
Number of non-glycine and non-proline residues	330	100.0%
Number of end-residues (excl. Gly and Pro)	30	
Number of glycine residues (shown as triangles)	30	
Number of proline residues	0	

Total number of residues	390	

PROCHECK

Ramachandran Plot

MODEL 3

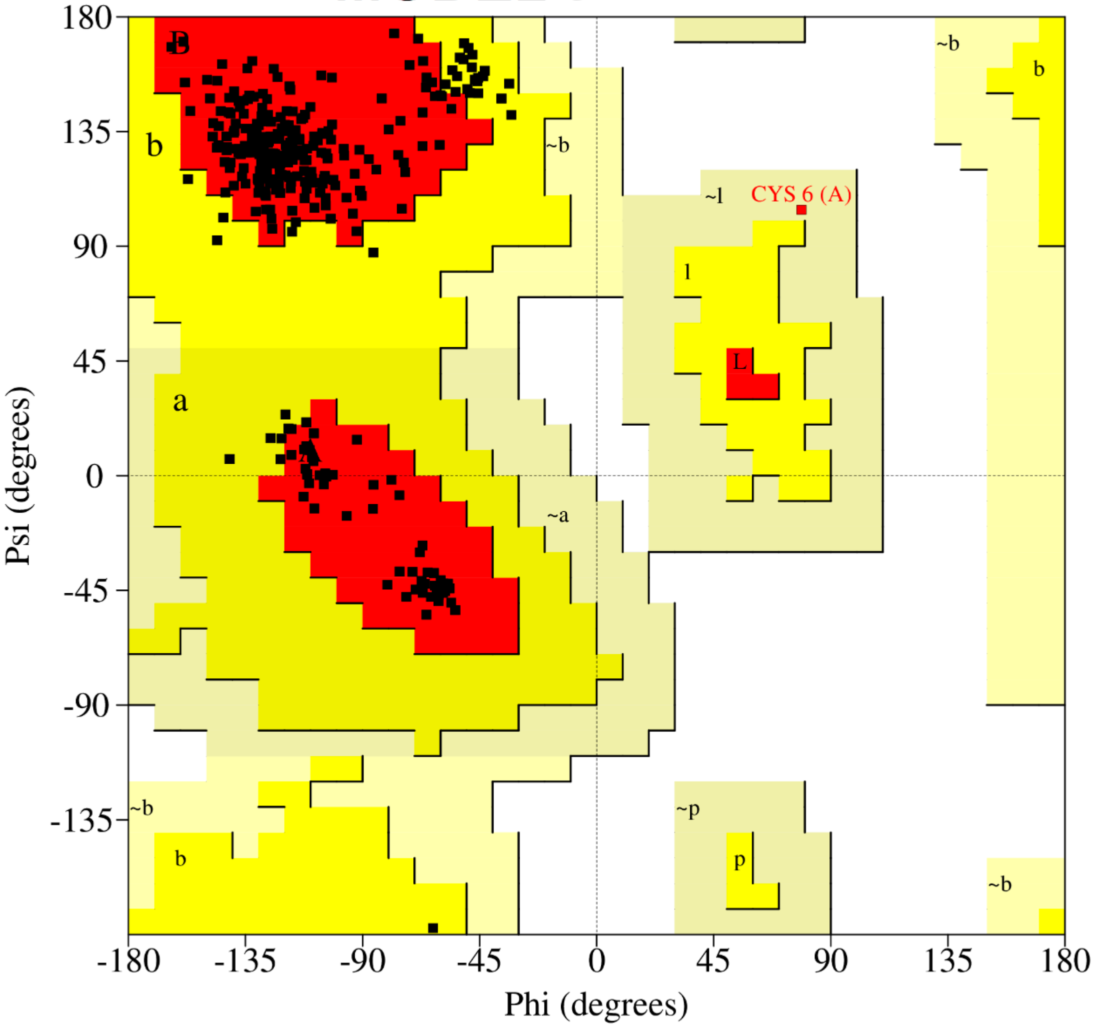

Plot statistics

Residues in most favoured regions [A,B,L]	294	89.1%
Residues in additional allowed regions [a,b,l,p]	35	10.6%
Residues in generously allowed regions [~a,~b,~l,~p]	1	0.3%
Residues in disallowed regions	0	0.0%
	----	------
Number of non-glycine and non-proline residues	330	100.0%
Number of end-residues (excl. Gly and Pro)	30	
Number of glycine residues (shown as triangles)	30	
Number of proline residues	0	

Total number of residues	390	

PROCHECK

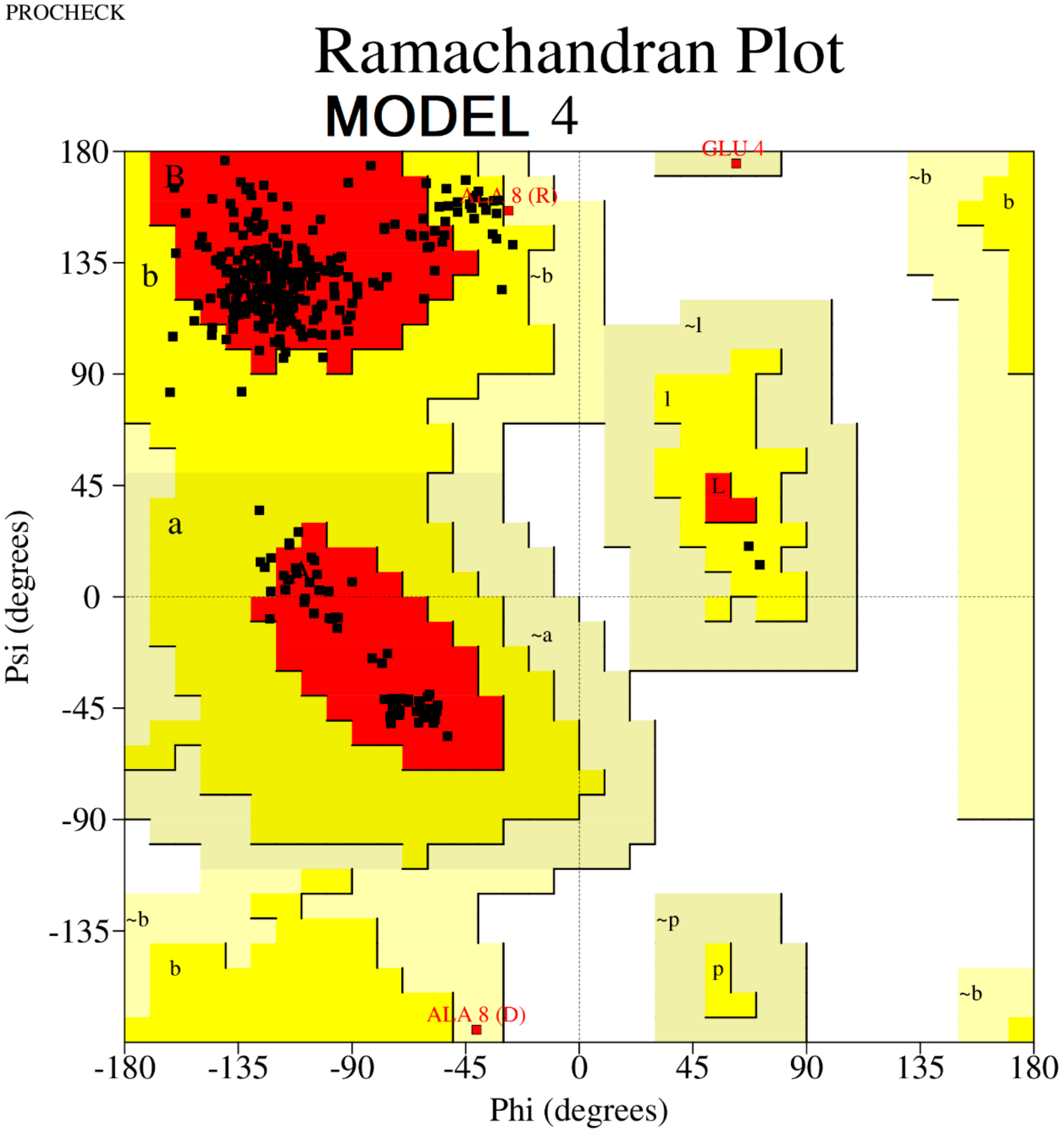

Plot statistics

Residues in most favoured regions [A,B,L]	285	86.4%
Residues in additional allowed regions [a,b,l,p]	42	12.7%
Residues in generously allowed regions [~a,~b,~l,~p]	3	0.9%
Residues in disallowed regions	0	0.0%
	----	------
Number of non-glycine and non-proline residues	330	100.0%
Number of end-residues (excl. Gly and Pro)	30	
Number of glycine residues (shown as triangles)	30	
Number of proline residues	0	

Total number of residues	390	

PROCHECK

Ramachandran Plot

MODEL 5

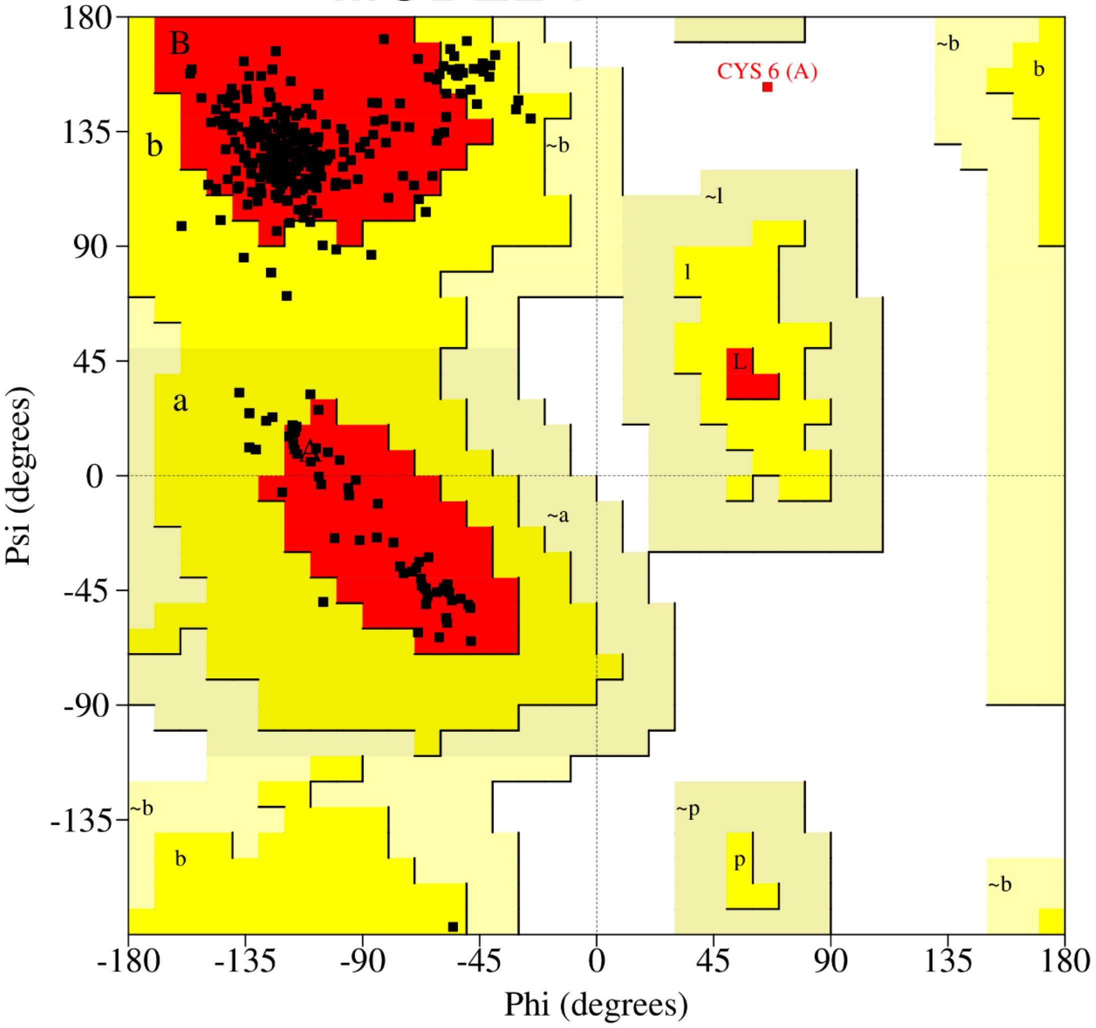

Plot statistics

Residues in most favoured regions [A,B,L]	288	87.3%
Residues in additional allowed regions [a,b,l,p]	41	12.4%
Residues in generously allowed regions [~a,~b,~l,~p]	0	0.0%
Residues in disallowed regions	1	0.3%
	----	------
Number of non-glycine and non-proline residues	330	100.0%
Number of end-residues (excl. Gly and Pro)	30	
Number of glycine residues (shown as triangles)	30	
Number of proline residues	0	

Total number of residues	390	

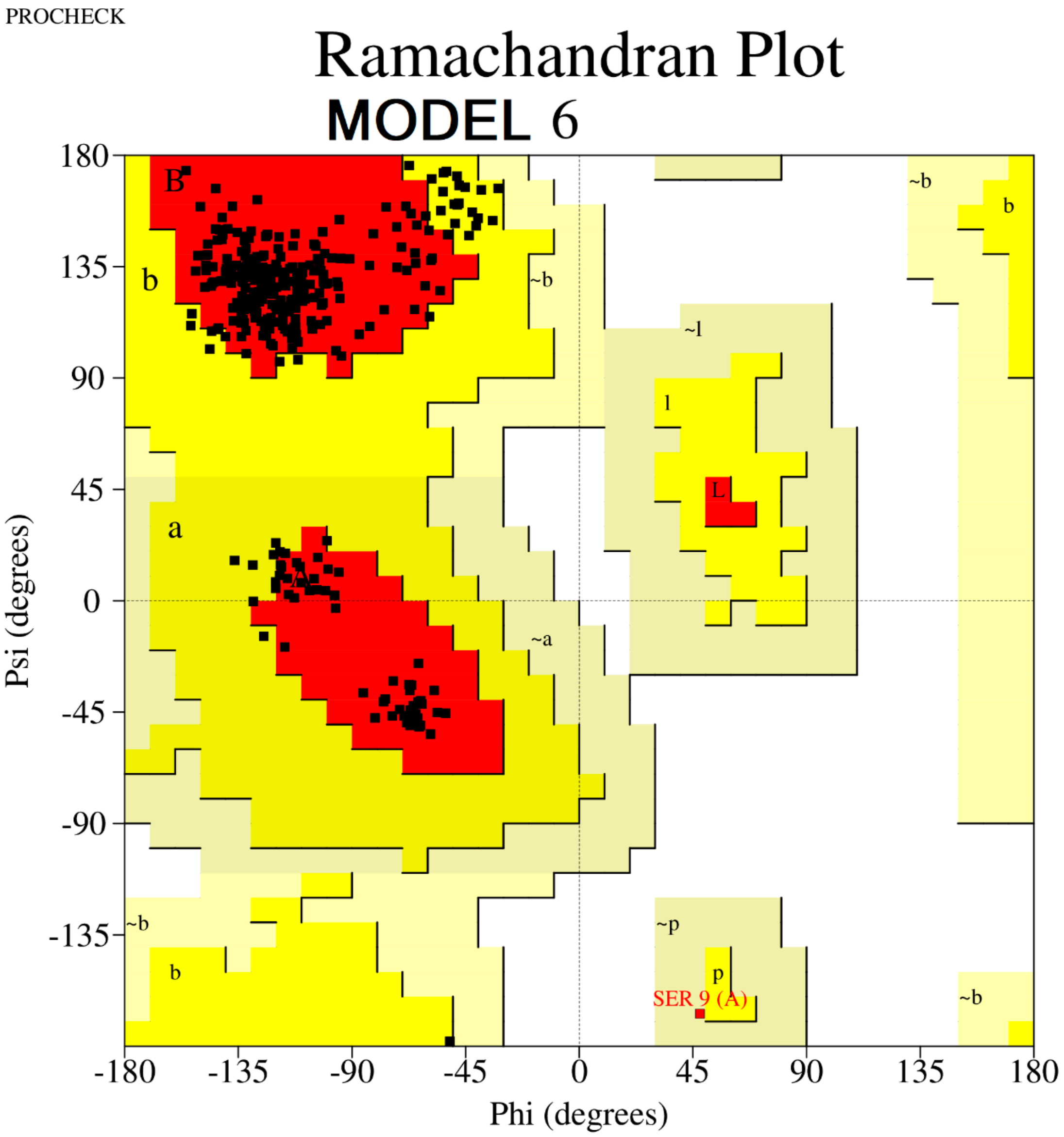

Plot statistics

Residues in most favoured regions [A,B,L]	290	87.9%
Residues in additional allowed regions [a,b,l,p]	39	11.8%
Residues in generously allowed regions [~a,~b,~l,~p]	1	0.3%
Residues in disallowed regions	0	0.0%
	----	------
Number of non-glycine and non-proline residues	330	100.0%
Number of end-residues (excl. Gly and Pro)	30	
Number of glycine residues (shown as triangles)	30	
Number of proline residues	0	

Total number of residues	390	

PROCHECK

Ramachandran Plot

MODEL 7

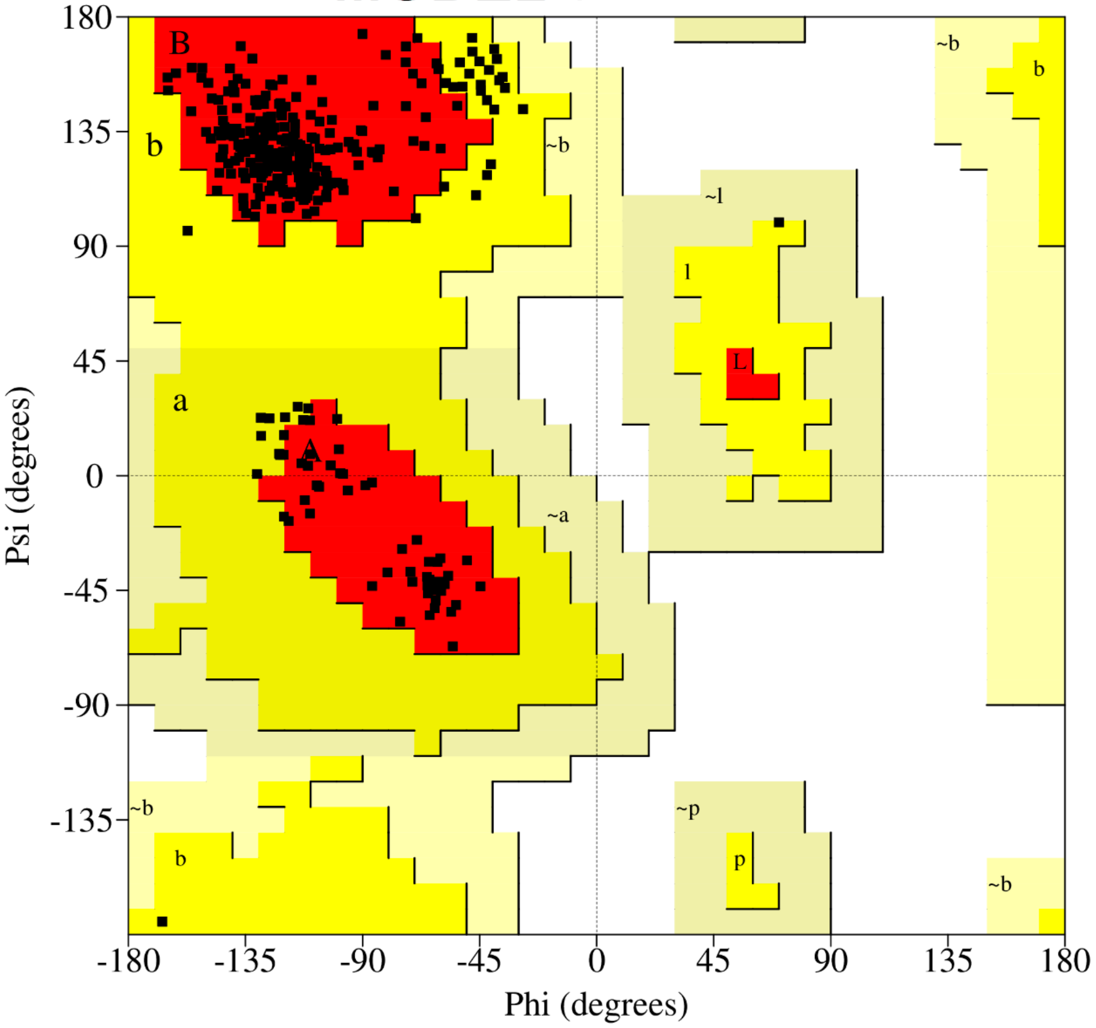

Plot statistics

Residues in most favoured regions [A,B,L]	287	87.0%
Residues in additional allowed regions [a,b,l,p]	43	13.0%
Residues in generously allowed regions [~a,~b,~l,~p]	0	0.0%
Residues in disallowed regions	0	0.0%
	----	------
Number of non-glycine and non-proline residues	330	100.0%
Number of end-residues (excl. Gly and Pro)	30	
Number of glycine residues (shown as triangles)	30	
Number of proline residues	0	

Total number of residues	390	

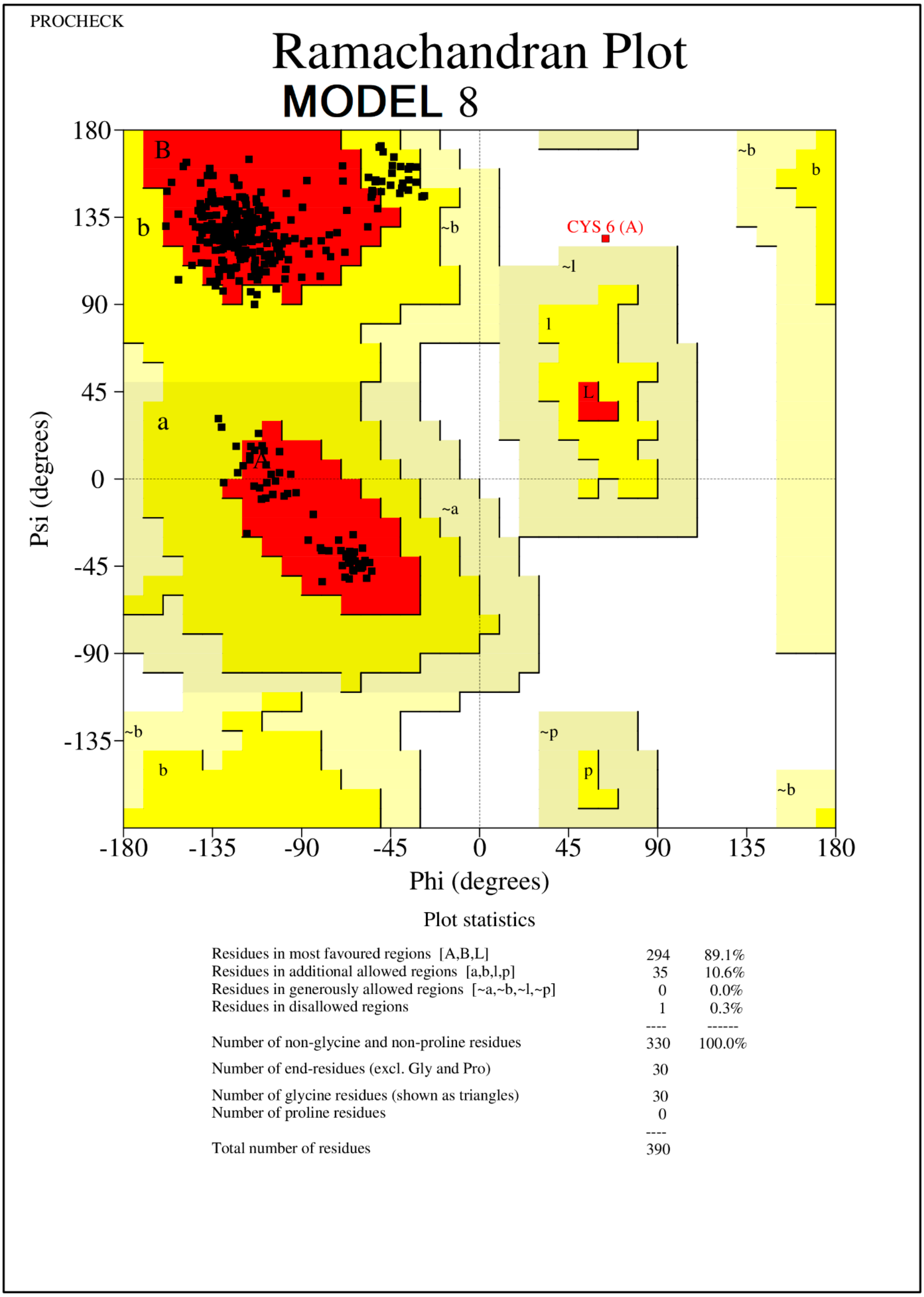

Figure S4. Ramachandran plots created using 8 predicted models of amyloid fibrils using PROCHECK program [1].

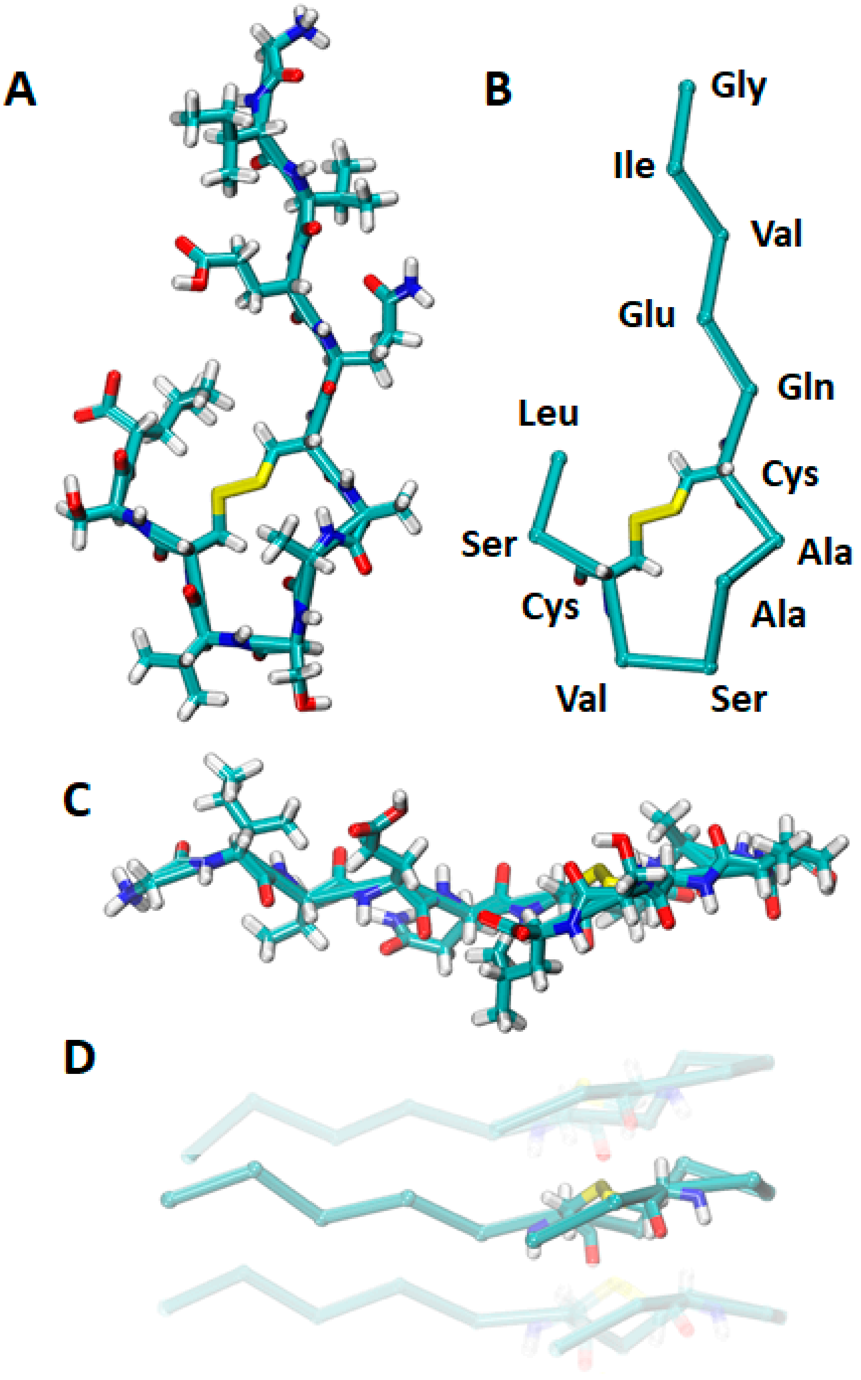

Figure S5. Structure of a single $ACC_{1\text{-}13}$ monomer extracted from the model of amyloid fibril. Panels A and B show single monomer chain in all-atom representation and C-alpha trace representation respectively (view along the long axis of the fibril). Panels C and D show single monomer in all-atom representation and the three adjacent monomer chains in C-alpha trace representation respectively (side view of amyloid fibril). Disulfide bond between Cys6 and Cys11 is shown in color yellow.

REFERENCES

1. Laskowski, R.A.; MacArthur, M.W.; Moss, D.S., and Thornton, J.M., *PROCHECK: a program to check the stereochemical quality of protein structures.* Journal of applied crystallography, 1993. **26**(2): p. 283-291.
